# Understanding the role of mask-wearing during COVID-19 on the island of Ireland

**DOI:** 10.1098/rsos.221540

**Published:** 2023-07-19

**Authors:** Nicola Fitz-Simon, John Ferguson, Alberto Alvarez-Iglesias, Mircea T. Sofonea, Tsukushi Kamiya

**Affiliations:** ^1^ School of Mathematical and Statistical Sciences, University of Galway, Galway, Republic of Ireland; ^2^ HRB Clinical Research Facility, University of Galway, Galway, Republic of Ireland; ^3^ MIVEGEC, Univ. Montpellier, CNRS, IRD, Montpellier, France; ^4^ Center for Interdisciplinary Research in Biology (CIRB), Collège de France, CNRS, INSERM, Université PSL, Paris, France

**Keywords:** COVID-19, mask-wearing, hospitalizations, counterfactuals, Ireland

## Abstract

Non-pharmaceutical interventions have played a key role in managing the COVID-19 pandemic, but it is challenging to estimate their impacts on disease spread and outcomes. On the island of Ireland, population mobility restrictions were imposed during the first wave, but mask-wearing was not mandated until about six months into the pandemic. We use data on mask-wearing, mobility, and season, over the first year of the pandemic to predict independently the weekly infectious contact estimated by an epidemiological model. Using our models, we make counterfactual predictions of infectious contact, and ensuing hospitalizations, under a hypothetical intervention where 90% of the population wore masks from the beginning of community spread until the dates of the mask mandates. Over periods including the first wave of the pandemic, there were 1601 hospitalizations with COVID-19 in Northern Ireland and 1521 in the Republic of Ireland. Under the counterfactual mask-wearing scenario, we estimate 512 (95% CI 400, 730) and 344 (95% CI 266, 526) hospitalizations in the respective jurisdictions during the same periods. This could be partly due to other factors that were also changing over time.

## 1. Introduction

Non-pharmaceutical interventions (NPIs) are actions taken by individuals or communities that aim to reduce infectious contacts between susceptible and infectious people during an epidemic [1]. Often governments may mandate such actions. Examples include reducing the number of physical contacts (e.g. closure of schools, workplaces, commercial establishments, roads and public transit; restriction of movement; cancellation of public events; maintenance of physical distances in public), reducing the chance of infection upon contact (e.g. mandatory use of personal protective equipment), and identifying and isolating those that may be infected (e.g. contact tracing and digital surveillance). By slowing the surge of infection, communities are afforded an opportunity to reduce infection-induced mortality and morbidity, alleviate health care burden and wait out an epidemic until pharmaceutical solutions (i.e. treatment and vaccines) become available. Public health policies to reduce the mixing of susceptible and infectious people have been instrumental in historical outbreaks, including during the 1918 influenza pandemic where rapid implementation of NPI mandates was crucial for reducing excess death in the USA [2].

Owing to the rapid spread of SARS-CoV-2 and the initial lack of effective therapy, NPIs have been central to managing the COVID-19 pandemic globally. NPIs such as staying at home and mask-wearing, both of which are often mandated, can reduce the reproductive number (i.e. the average number of secondary cases per infectious host) and sometimes bring it below 1, thereby halting the growth of SARS-CoV-2 infections in a population [3,4]. Reducing inter-individual infectious contact has also been shown to reduce the relative advantage of highly transmissible variants [5]. Even after a widespread rollout of vaccines, understanding the effectiveness of NPIs and the role of mandating them remains pertinent as vaccination alone is unlikely to put an end to the pandemic [6] and vaccination and NPI can synergistically reduce SARS-CoV-2 transmission [7]. Despite their public health benefits, social distancing measures have been shown to incur high costs in several domains, including in the economy [8], mental health [9] and civil liberty [10]. Thus, it is crucial to quantify the effectiveness of interventions to achieve desired public health outcomes and improve policy transparency and public engagement.

Mechanistic epidemiological models have been widely applied to study the dynamics of SARS-CoV-2, and to make predictions of clinical outcomes under alternative scenarios (e.g. an assumed decrease in physical contact [11]). Furthermore, fitting a mathematical model to data on observed processes such as reported cases and hospital admissions allows estimation of inter-individual infectious contact [12,13]. In a companion paper, we have estimated longitudinal infectious contact ratios for SARS-CoV-2 in Northern Ireland (NI) and the Republic of Ireland (ROI) using a multi-strain compartmental model [14]. While mechanistic epidemiological models are not primarily intended for the estimation of the effects of NPI, there has been some recent work on incorporating effect estimation in a mechanistic or semi-mechanistic modelling framework. For example, the Institute for Health Metrics and Evaluation (IHME) COVID-19 Modelling Team used compartmental epidemiological models to estimate time-varying rates of infection of susceptibles, and subsequently used regression models to predict these rates in terms of NPI variables; this allowed them to make forecasts of infection rates, hence mortality and morbidity outcomes [15]. Other relevant approaches that link NPI to disease transmission include those that incorporate digital mobility data in a (semi-)mechanistic disease model (e.g. [16,17]). An alternative approach involves structural equations that model potential outcomes such as deaths or cases in terms of interventions of interest (e.g. [18–20]).

On the island of Ireland, the first diagnosed case of COVID-19 returned to Northern Ireland from Italy on 17 February 2020 [21]. The epidemiological development of the pandemic in Northern Ireland and the Republic of Ireland was similar with regards its timing and extent. The initial wave of the pandemic peaked towards the end of April 2020. Strict lockdowns remained in place over early summer, with gradual easing of restrictions later in the summer. Meanwhile, in both jurisdictions, mask-wearing in public places was initially discouraged, and was not mandated in public places until August 2020. A second smaller wave occurred in November; lifting of many restrictions ahead of Christmas preceded the third and largest wave of the pandemic around Christmas 2020. We describe the epidemiological development of the pandemic on the island of Ireland in more detail in [14]. In the ROI, over 13 000 people were hospitalized with COVID-19 and 4600 died in the first year up to March 2021 [22].

Our aim in this paper is to investigate what might have happened with regards COVID-19 outcomes under a counterfactual scenario wherein most people wore masks in public during the early part of the COVID-19 pandemic in Ireland, including the first wave. Our study rationale leverages the reality that public health advice discouraged population mask-wearing during the early part of the pandemic, and few people wore masks in public places during the first wave [23]. Thus, infectious contact during the first few months of the pandemic in Ireland was little mitigated by population mask-wearing, whereas during the latter half of 2020 mask-wearing was mandated. However, we were not able to simply study the effects of mask-wearing mandates, as on the island of Ireland behavioural change preceded mandates. Thus to achieve our aim, our objectives were: (1) to compile and describe data on actions that aimed to reduce the potential for inter-individual infectious contact on the island of Ireland, specifically data on behavioural changes such as mobility patterns and mask-wearing prevalence; (2) via regression models, to use these data to independently predict weekly infectious contact ratios we previously estimated for both jurisdictions on the island of Ireland [14]; and (3) to make counterfactual predictions of infectious contact and ensuing hospitalizations under a hypothetical intervention where 90% of the population were wearing masks in public during the first six months of the pandemic in Ireland.

## 2. Methods

In this section, we describe in detail the data we used and all models we fitted as part of our analyses. For transparency, and to facilitate other researchers who wish to apply similar methods in different settings, we have also provided all our data and code at https://github.com/Nicola-Fitz/masks, as detailed in the Data accessibility section below.

### 2.1. The infectious contact ratio

The infectious contact rate at any point in time is a measure of the potential for inter-individual infectious contact; we estimated this as a ratio relative to its value at the beginning of community spread in Ireland, which we previously estimated to be around 5 March 2020 [14]. To estimate longitudinal infectious contact ratios, we used a discrete-time compartmental model that incorporates multiple virus strains with different transmissibilities. We estimated these by week for both NI and the ROI over the first year of the pandemic (5 March 2020–28 February 2021), fitting the model to longitudinal data on hospital admissions with COVID-19 and proportion of B.1.1.7, or Alpha, strain cases in each jurisdiction. To parametrize the model, distributions of the time from infection to transmission, and from infection to hospitalization for hospitalized cases, were based on published evidence [24–26]. As our epidemiological model allowed for durations in each disease compartment informed by evidence, we were able to model accurately the short-term dynamics of the disease, meaning that the infectious contact ratios we estimated reflect short-term behaviour. This makes the infectious contact ratio a suitable outcome for modelling in terms of predictors that also changed in short time frames, such as mobility. Our epidemiological model, model fitting in a Bayesian framework, and results are described in detail in our companion paper [14]. For subsequent analyses in the current paper, we took a single replicable draw from the posterior distribution of estimated weekly infectious contact ratios over the first year of the pandemic for each jurisdiction, and used the posterior medians as our measure of weekly infectious contact.

### 2.2. Data to predict infectious contact ratio

The governments of ROI and NI carried out a series of public health interventions to curtail the transmission of SARS-CoV-2. We used publicly available sources to compile data on timing and type of policy restrictions in NI and the ROI between March 2020 and March 2021; further detail on these is available in [14]. NI followed a different approach to the ROI in introducing and easing restrictions; the UK has a COVID-19 alert system with five levels of alert depending on the level of COVID-19 in the community [27], whereas the ROI defined levels of restriction to mitigate the impact of the virus [28].

As a measure of the extent of physical contact between people, we extracted data from the COVID-19 Google Community Mobility reports for NI and the ROI. These provide six population mobility streams relative to a pre-pandemic baseline [29]. We computed a 7-day moving average of three streams, namely workplaces, transit stations and retail and recreation, as these are thought to be the most appropriate to reflect relevant mobility patterns ([30] used an average of these three streams plus grocery and pharmacy to reflect relevant movement patterns outside the home). As the mobility data for NI were reported by local government district, we calculated an overall seven-day moving average weighted by the population of each district. Further detail on the mobility streams is provided in electronic supplementary material, S1.

We gathered data on the population proportion reporting to be wearing masks in public, and other behavioural data, from behavioural surveys published by the Irish Department of Health, and the Northern Ireland Statistics Research Agency (NISRA) [31,32]. These were based on regular cross-sectional behavioural surveys of 1200 people in NI and 1500–2000 in the ROI; the NI samples were weighted for household size and non-response and the ROI samples weighted to demographics [31,33]. While these behavioural surveys were done from March 2020 in ROI and April 2020 in NI, questions about mask use in public places were only included from 4 May in the ROI, and from 17 June in NI. We assumed that 1% of the population was wearing masks in public at the beginning of the epidemic on the island of Ireland, and fitted a binomial regression model with a spline smooth for time to predict proportion wearing masks on dates not reported in the surveys.

The behavioural surveys did not include information on the type of masks worn; however, during the first year of the pandemic, public health advice and mandates were to wear cloth face coverings (as opposed to medical grade or respirator masks) [34].

Seasonality may play a role in virus transmission, as people are more likely to be in indoor settings more conducive to disease spread during colder months [35]. To define seasons, we used Met Éireann daily weather data from Dublin airport [36]. We calculated 7-day running means of the median daily temperature, and defined the summer season to start when this temperature first exceeded 10.5°C, and to end when it first dropped below this value. The summer season thus defined ran from 17 May until 21 September. Using warmer temperatures to define the summer period aims to identify a period when people are less likely to be indoors.

### 2.3. Regression models

In this section, we describe our linear regression model to predict infectious contact. We initially described the relationship between the estimated weekly infectious contact ratios, the overall Google mobility stream (modelled linearly), and longitudinal mask-wearing proportion in each jurisdiction. We fitted a linear regression model with response the natural log of the weekly contact ratios and predictors the jurisdiction (NI or ROI), the season (summer or winter), the Google mobility stream and smoothed proportion mask-wearing at the mid-point of each week. We assumed the effect of proportion mask-wearing conditional on the other predictors to be linear and the same in each jurisdiction. We included an interaction term between season and proportion mask-wearing to allow for a different effect of mask-wearing in the winter when people are more likely to be indoors. We included an interaction term between mobility and mask wearing to allow for a change in the effects of each as the level of the other changes. For instance, the effect of increasing mobility may be attenuated when most people are wearing masks compared to when few people are wearing masks. To explore assumptions of linearity, we also fitted a model including spline smooths for the effect of the proportion wearing masks on the response. We calculated goodness-of-fit statistics for the predictive models.

Our regression model assumes that the responses are independent, which is not the case, as they are correlated over time due to the smoothing described in [14]. Therefore, we estimated heteroskedasticity and autocorrelation consistent Newey–West standard errors for the parameter estimates, hence adjusted the confidence intervals for the predicted responses from separate models for each jurisdiction [37].

### 2.4. Counterfactual scenarios

We compared the observed responses with predicted responses under a hypothetical intervention where 90% of the population were wearing masks during the early months of the pandemic. In effect, we leveraged observed data from late 2020, when over 90% of the population were wearing masks, to estimate the counterfactual scenario.

To relate the counterfactual infectious contact to disease outcomes, we returned to our multi-strain epidemiological model to predict hospital admissions from the counterfactual infectious contact ratio. We compared the sum of predicted hospital admissions under the intervention with observed hospital admissions up to the date of the mask mandates (10 August 2020 in both jurisdictions). As a check, we compared the number of hospitalizations predicted by the epidemiological model under no intervention with the observed number of hospitalizations for consistency.

We estimated confidence intervals for the difference in hospital admissions for the counterfactual scenario versus reality in each jurisdiction by bootstrapping the process of estimating regression models, predicting the outcome under the counterfactual scenario, and using this predicted outcome (the counterfactual infectious contact ratio) to predict counterfactual hospital admissions from the multi-strain epidemiological model. We ran 10 000 bootstrap replications, and estimated bias-corrected and accelerated (BCa) confidence intervals.

We considered the possible impact of confounding by including potential measured confounding variables that were also changing over time in the regression models, and considering the possible impact of unmeasured confounding.

## 3. Results

### 3.1. Estimated infectious contact and mobility

**Figure 1.**
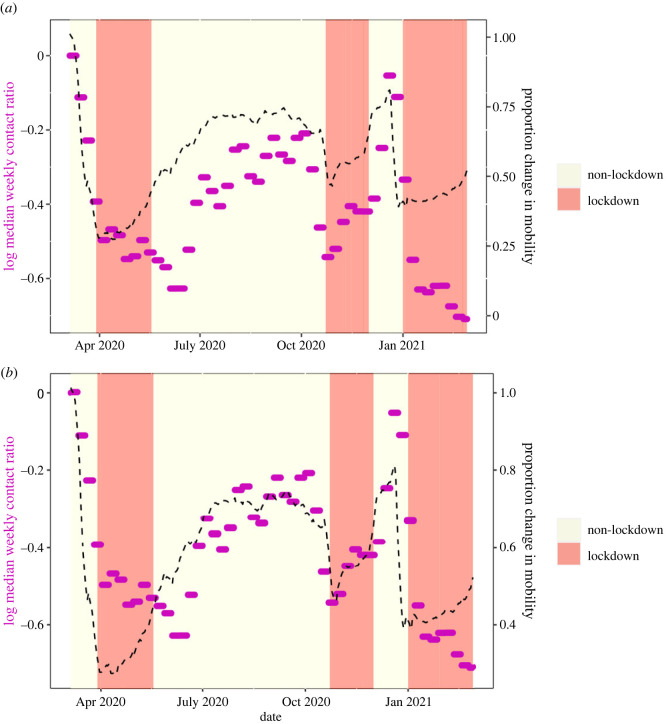
Restriction level, mobility and infectious contact ratios for the Republic of Ireland. Red shaded regions show periods of the highest restrictions (i.e. lockdown). Median weekly contact ratios are shown as purple dots. The averaged Google mobility stream is shown as a black dashed line. In (*a*), the mobility stream is scaled to coincide with contact ratios at the beginning of the period (up to mid-May). In (*b*), the mobility stream is scaled to coincide with period May–December.

**Figure 2.**
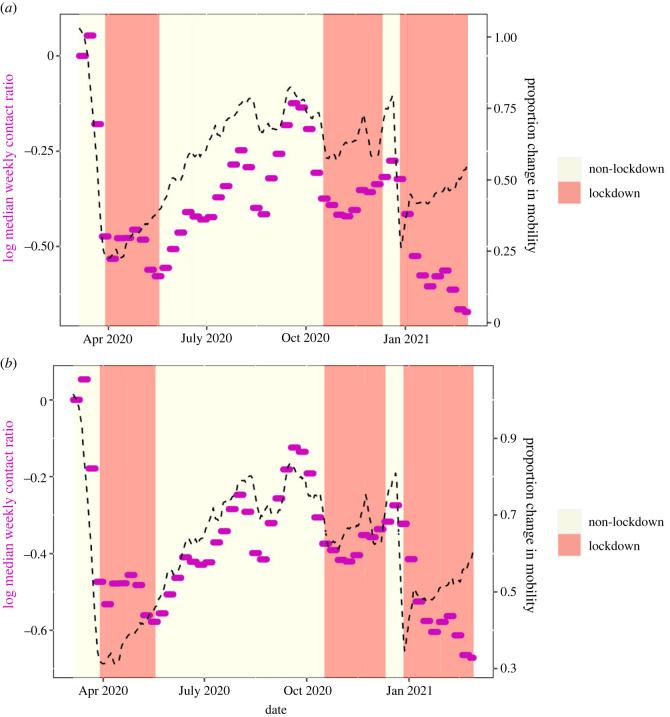
Restriction level, mobility and infectious contact ratios for Northern Ireland. Red shaded regions show periods of the highest restrictions. Median weekly contact ratios are shown as purple dots. The averaged Google mobility stream is shown as a black dashed line. In (*a*), the mobility stream is scaled to coincide with contact ratios at the beginning of the period (up to mid-May). In (*b*), the mobility stream is scaled to coincide with period May–December.

**Figure 3.**
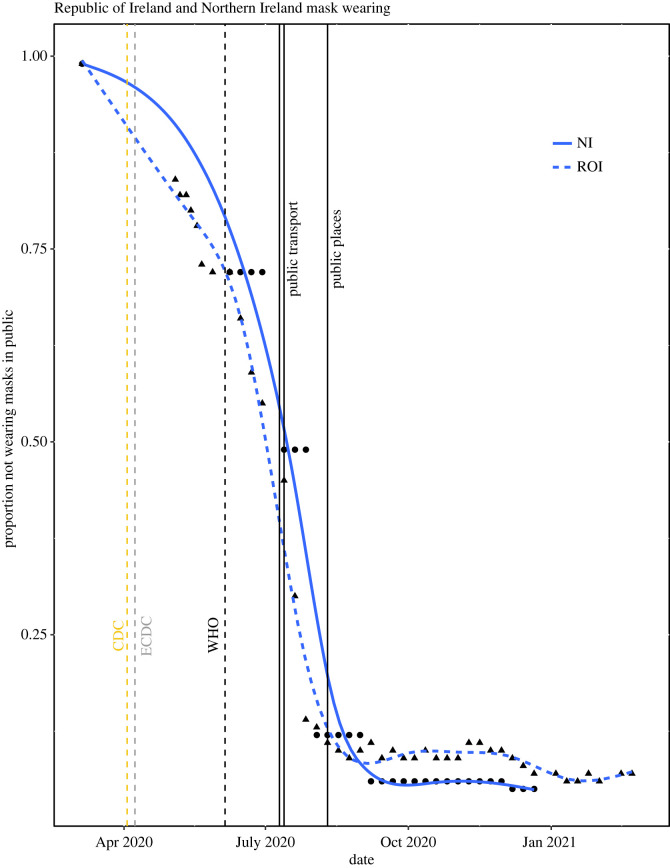
Reported use of face masks in public places on the island of Ireland, with spline smooths for each jurisdiction (NI circles, solid line; ROI triangles, dashed line). Dates of public health mandates to wear masks on public transport (10 June in NI, 13 June in ROI), and in all public places (10 August 2020 in both NI and ROI) are shown as black vertical lines. The dates on which major public health institutions changed their recommendations on mask-wearing for the general population from advising against wearing masks to wearing masks in indoor public places are shown as dashed vertical lines: US Centers for Disease Control and Prevention, CDC (3 April 2020), European Centre for Disease Prevention and Control, ECDC (8 April 2020), World Health Organisation, WHO (5 June 2020).

Infectious contact is likely affected by the extent of physical contact between people, which may be reflected by digital mobility data. To better understand how mobility behaviour affects infectious contact, we explored the relationship between the infectious contact ratio and the overall Google mobility data stream described above. We observed that the relationship between infectious contact and mobility shifted in early summer 2020 and again in January 2021 (figures 1 and 2). This finding mirrors that of Nouvellet *et al.* [30], who demonstrated a change in the relationship between mobility and the reproductive number in mid-2020 in many countries, which they attributed to the increased use of NPIs, such as contact tracing. We also note that the change in the relationship between infectious contact ratio and mobility in both NI and the ROI coincides with an increase in mask wearing (figure 3). A later change in the relationship, in January 2021 may be due to the beginning of the vaccination programme (and this effect appears larger in NI, where the vaccination roll out was faster; albeit few second doses were administered during the period we study [38,39]).

### 3.2. Mask mandates and mask-wearing behaviour

Google mobility data and our epidemiological model demonstrate that decreases in mobility (and infectious contact ratios) preceded lockdowns (figures 1 and 2). Change in behaviour ahead of government mandates is also evident from comparing mask mandates with level of self-reported mask-wearing in public places (figure 3). In both NI and ROI, around 90% of people reported wearing masks before the date of the national mask mandates on 10 August 2020. While early advice largely discouraged people from wearing masks (e.g. [40]), the US Center for Disease Control and Prevention changed their advice to recommend mask-wearing in public places on 3 April 2020 [41], closely followed by the European Centre for Disease Prevention and Control on 8 April 2020 [42]. While behavioural data on mask-wearing in public were only collected from May in the ROI and from June in NI, 16% of people in NI reported wearing masks in early May, and the proportion of people wearing masks increased more rapidly after the World Health Organisation changed its advice to recommend them on 5 May 2020 [43]. Taken together, global and international public health advice likely had a greater influence on the mask-wearing behaviour than the subsequent national mandates in both ROI and NI. Thus, we focus our attention on estimating the impacts of actual behavioural change, in particular mask-wearing, rather than the impacts of government mask-wearing mandates.

### 3.3. Regression models to predict infectious contact ratio

**Figure 4.**
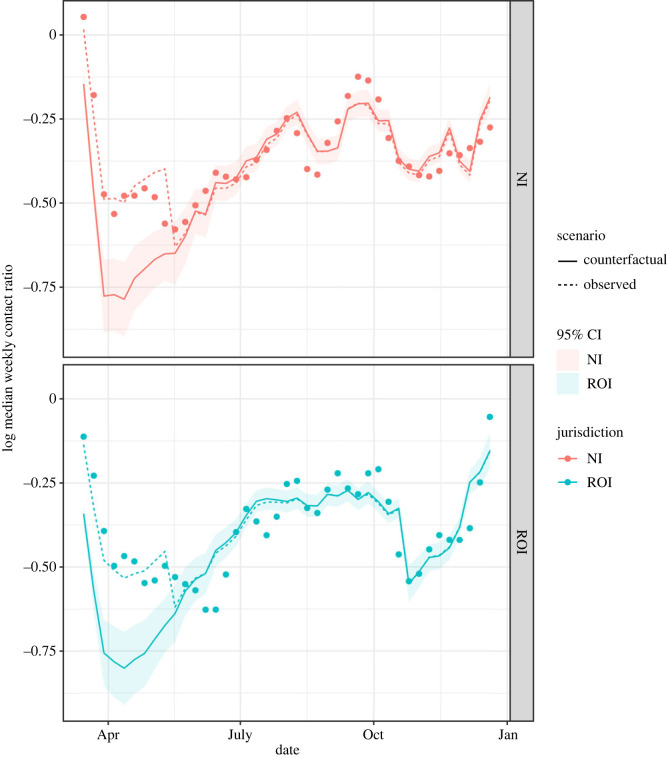
The dots show the log weekly infectious contact ratios. The dotted lines are the predicted log contact ratios from a linear regression model with predictors jurisdiction, season, average mobility and proportion of people wearing masks. The solid lines show predicted log contact ratios and 95% CIs under a hypothetical intervention where 90% of people wore masks throughout. In the hypothetical situation, mobility is assumed to remain as observed in reality.

We fit linear regression models for 2020 data only, as we have observed that the relationship between our response variable and mobility changes around the end of 2020, around the time the vaccination programme began, and it is possible this would have impacted on the potential for inter-individual infectious contact. We found that the regression model with a linear effect of proportion wearing masks predicts the log weekly contact ratios reasonably well (figure 4; broken lines); with AIC −208.6 and *R*^2^ 0.77. Parameter estimates for this model are given in electronic supplementary material, table S2. A spline smooth for mask-wearing provides a better fit with AIC −218.1 and *R*^2^ 0.81. However, the improvement is not vast, and the assumption of a linear relationship with log contact ratio is more consistent with other evidence about population mask-wearing [44]; we therefore assume a linear effect for the subsequent counterfactual analysis.

Newey–West heteroskedasticity and autocorrelation consistent sandwich estimators for the parameter variances in regression models for each jurisdiction separately gave slightly decreased standard errors of most parameter estimates [37]. This deflated pointwise confidence intervals for predicted responses slightly in some regions, and inflated them slightly in others (see electronic supplementary material, S3). As the impact on the confidence bands for predicted log infectious contact was small, we ignored the non-independence of responses when estimating confidence intervals for the number of hospitalizations saved.

### 3.4. Counterfactual scenarios

We used the linear regression model to predict log infectious contact under the counterfactual scenario that 90% of the population were wearing masks during the early part of the epidemic on the island of Ireland up to the date of the mask mandates, while mobility remained as observed in reality. The counterfactual scenario predicts a marked decrease in infectious contact during the first wave in both jurisdictions compared with that observed in reality (figure 4; solid lines). As described in the Methods section, the regression model assumes the same linear effect of increasing the population proportion wearing masks on the log contact ratio in NI and ROI, conditional on season and mobility. In addition, in the two jurisdictions the weekly pattern of self-reported mask-wearing ahead of the mask wearing mandate was very similar (figure 3) so that in the counterfactual scenario, increasing the proportion wearing masks in each jurisdiction to 90% requires a similar increase in proportion for each jurisdiction at each week throughout this period. Thus, the predicted counterfactual log contact ratios are similar in each jurisdiction.

Electronic supplementary material, S4, shows a contour plot of observed data together with the model predictions as a function of mobility and mask-wearing for summer and winter separately. We have considered an intervention on mask-wearing for which we have a reasonable amount of observed data to estimate the counterfactual scenario. However, this does involve some extrapolation beyond the range of the observed data to a region of high mask-wearing and very low mobility.

While there were in total 1601 observed COVID-19 hospitalizations in NI from 12 March until the date of mask mandate on 10 August (1690.3 predicted from the epidemiological model), we predicted 512.0 (95% CI 399.6, 729.8) under the counterfactual scenario—that is, 1089 (871, 1201) fewer hospitalizations. Daily hospital admissions in the ROI were not publicly available before 3 April, but there were 1521 admissions between this date and the mask mandate (1531.8 predicted from the epidemiological model), while our counterfactual mask-wearing scenario predicted 343.8 (95% CI 266.2, 526.2) between 3 April and 10 August—that is, 1177 (995, 1255) fewer hospitalizations.

**Figure 5.**
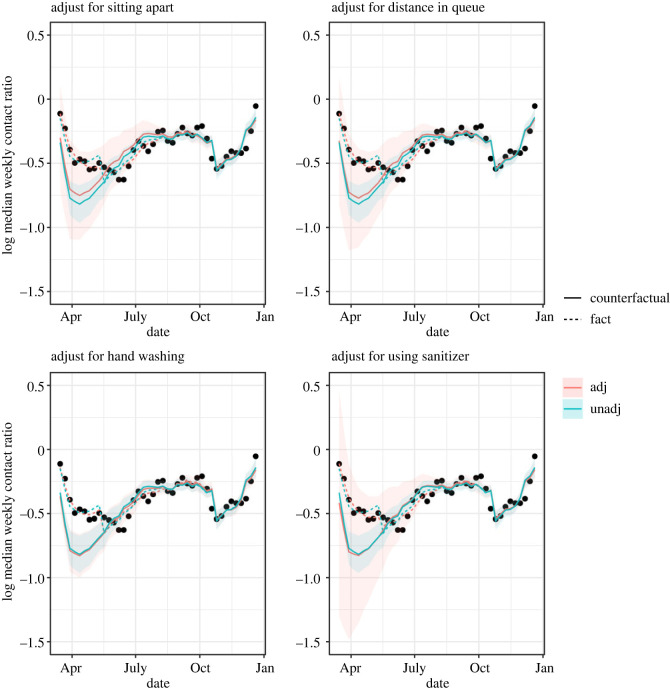
Republic of Ireland. The dots show the log weekly contact ratios. The dotted lines are the predicted log contact ratios from a linear regression model with predictors season, average mobility, proportion of people wearing masks, and the NPI variable of the title. The solid lines show predicted log contact ratios and 95% CIs under a hypothetical intervention where 90% of people wore masks throughout. In the hypothetical situations, mobility and the other NPI variable are assumed to remain as observed in reality.

The behavioural data reported by the Irish Department of Health also included information on other NPI measures: the proportions of people reporting to be sitting further apart from others, and distancing when in a queue, compared to before the pandemic, and the proportions of people washing their hands, and using sanitizer [31]. We estimated the predicted proportions of these NPI variables from binomial regression models with spline smooth for time, as we did to estimate the predicted proportion wearing face masks at intermediate dates. As a sensitivity analysis for confounding by other NPI variables, we fit regression models for the log weekly contact for the ROI as above, and also adjusting for predicted value of each NPI variable, and an interaction between mask-wearing and the other NPI variable to reflect the assumption that the effects of multiple NPIs are not additive. Figure 5 shows for each NPI variable that the predicted log contact ratio under the 90% mask-wearing intervention with adjustment for the NPI variable was only slightly different from that without each adjustment. However, pointwise 95% confidence intervals were greatly inflated for each adjustment apart from hand-washing, so that the actual log contact ratios fell within the adjusted confidence bands. The proportion hand washing was high and fairly constant throughout the period considered; the other three NPI variables were increasing at the same time as mask-wearing, though none to the same extent. The inflation of pointwise confidence intervals could be due to co-linearity of the predictor variables.

## 4. Discussion

Most attempts to estimate the effects of NPIs on COVID-19 outcomes—either by incorporating effect estimation in a mechanistic epidemiological model, or by using structural equations models—have been in a US setting (e.g. [15–20]). In the ROI, compartmental models have been used by several research groups to understand the dynamics of the virus, make forecasts of outcomes under various scenarios and assess economic impacts of policy restrictions [45–48]. Several UK studies also included NI data to understand regional SARS-CoV-2 dynamics [49,50].

An important difference between the infectious contact parameter and time varying reproductive number is that the former is unaffected by changes in virus transmissibility, which is modelled independently in our approach [14]. This separation is especially useful to eliminate bias due to concurrent changes in NPIs and virus transmissibility. By contrast, biases may result from estimating the effect of NPI on the time-varying reproductive number during transition periods where this mixture in the population is changing. It is however possible that mask-wearing is less effective for more transmissible variants [51], in which case we may have underestimated contact ratios and hospitalizations in the counterfactual scenarios; however, as the alpha strain only became dominant at the end of 2020, the potential for bias is slight.

A further advantage of using the infectious contact ratio as the outcome in our investigation of the effect of population mask-wearing is that the proportion wearing masks may affect the infectious contact ratio at the current week, but should not affect the infectious contact ratio at other (future) weeks. In infectious disease studies, estimating the effect of interventions is challenging when the intervention on one unit can affect the outcome of another, as could be the case if the outcome were the level of infection in the community. Our use of the infectious contact ratio bypasses this problem of interference, with the caveat that there is autocorrelation between successive estimated contact ratios due to the smoothing described in [14]. Nonetheless, we also found in that study that the bias was small and unlikely to have a substantive effect on our results.

In the regression models, we have treated the observed responses as independent, but as noted there is some dependence in estimated weekly infectious contact ratios over time. Calculating Newey–West heteroskedasticity and autocorrelation consistent sandwich estimators for the parameter variance matrices in the regression models deflated the confidence intervals for the predicted responses slightly in some cases, and increased them slightly in others, but the difference to confidence intervals for predicted responses assuming independence was small.

A limitation of this study is that in estimating the effect of mask-wearing on the infectious contact ratio, we have not adjusted for all potential sources of confounding. For example, other actions to mitigate the spread of the virus may also have been changing at the same time as mask wearing, and may be partly responsible for the effect we attribute to wearing masks. Behavioural survey data from the Irish Department of Health and NISRA indicate that many of the relevant behaviours that could bias the effect remained fairly constant throughout (for example, hand washing, which was high from early during the period considered), or increased to a smaller extent than mask-wearing (e.g. self-reported social distancing) [31,32]. When we included other NPI variables in the regression model, counterfactual predictions were little changed, but confidence intervals were inflated; this could be due to co-linearity. In addition, over time people could have been becoming better at taking various different actions to mitigate the spread of the virus, and we have no measure of improvement in implementation of NPIs. As for any observational study, unobserved confounding is a major limitation in estimating causal effects.

As we did not observe many combinations of mask-wearing and mobility, especially when mobility was very low, the counterfactual scenario involves some model extrapolation. This influenced our modelling assumption that the effects of mask-wearing on log contact ratio are linear rather than using the better fitting spline smooth; the latter is consistent with the evidence that the population proportion not wearing masks has a multiplicative effect on the reproductive number [44].

In summary, we have predicted infectious contact from season, population mask-wearing, and mobility, in the specific context of the island of Ireland. Our study corroborates the body of the literature demonstrating the effectiveness of mask-wearing [52,53]. Our findings relate mainly to the use of cloth face coverings and suggests these can still mitigate community spread of SARS-CoV-2, even though they are acknowledged as not as effective as other types of mask.

We found that increasing mask-wearing to 90% throughout early 2020 would have decreased inter-individual infectious contact during the first wave, and, in consequence, led to substantially fewer predicted hospitalizations with COVID-19. The disease outcomes that most reliably captured the spread of the disease in the community on the island of Ireland were longitudinal hospital admissions with COVID-19; we used these to estimate weekly infectious contact ratio via our epidemiological model. It was simply a case of reversing the epidemiological model to predict hospitalizations from the counterfactual infectious contact ratios. Other disease outcomes of interest include cases, intensive care admissions and deaths, none of which we attempted to predict in the current analysis, but could be predicted from an extended version of the epidemiological model and under further assumptions. While we did not quantify these outcomes, a reduction in infectious contact also implies a reduction in cases, intensive care admissions, and deaths. Our findings suggest that, all else being equal, early population adoption of mask-wearing in public places could have slowed the initial spread of SARS-CoV-2 in Ireland, with possible implications for disease outcomes including but not limited to hospitalizations during the first wave. Clearly, this has implications for the public health response to future respiratory virus pandemics. An interesting question is around the longer term implications for the spread of the disease and outcomes on the island of Ireland, had population mask-wearing been adopted at the beginning of the pandemic; however, this is beyond the scope of our current analysis. While our analysis provides some evidence, further research is needed to better understand the epidemiological and public health impacts of population mask-wearing.

The effect we estimated is likely to have been at least partly due to changes in other factors that mitigate the spread of the virus and were also changing over time, and may be better interpreted as an effect of many accumulated actions to prevent the spread of the virus that were changing over time. Mask-wearing was the one NPI for which international public health bodies and Western governments changed their recommendations; as we have previously noted, early advice strongly discouraged mask-wearing, even claiming this put mask-wearers at higher risk of becoming infected; however by the summer of 2020 the consensus was that mask-wearing was beneficial. Changes in international and national public health recommendations about mask wearing likely played a role in the patterns of mask-wearing uptake, leading to very large increases in mask-wearing between March and August 2020; this is in contrast to data on other NPIs where the public health messaging was more consistent and changes in behaviour were not as large. It is unlikely that the smaller changes in other NPIs could account for the entire effect of mask-wearing we have reported.

## Data Availability

Code and datasets are available at https://github.com/Nicola-Fitz/masks. All datasets used in this research are publicly available. Data from the Google Covid-19 community mobility reports are at https://www.google.com/covid19/mobility/. Data on mask-wearing in Northern Ireland are at https://www.nisra.gov.uk/publications/nisra-coronavirus-covid-19-opinion-survey-previous-results. Data on mask-wearing and other behaviours in the Republic of Ireland are at https://www.gov.ie/en/collection/6b4401-view-the-amarach-public-opinion-survey/. Data on hospital admissions for Northern Ireland are from https://www.health-ni.gov.uk/articles/covid-19-daily-dashboard-updates, and for the Republic of Ireland from https://covid19ireland-geohive.hub.arcgis.com. Data on weather are from https://www.met.ie/climate/available-data/historical-data. The data are provided in electronic supplementary material [54].
